# A White Campion (*Silene latifolia*) floral expressed sequence tag (EST) library: annotation, EST-SSR characterization, transferability, and utility for comparative mapping

**DOI:** 10.1186/1471-2164-10-243

**Published:** 2009-05-25

**Authors:** Maria Domenica Moccia, Christine Oger-Desfeux, Gabriel AB Marais, Alex Widmer

**Affiliations:** 1 ETH Zurich, Institute of Integrative Biology (IBZ), Universitaetstr. 16, 8092 Zürich, Switzerland; 2 DTAMB/PRABI, IFR41, Université Lyon 1, Bâtiment Gregor Mendel, Villeurbanne, F-69622 cedex, France; 3 Université Lyon 1, CNRS, UMR5558; Laboratoire de Biométrie et Biologie évolutive, Villeurbanne, F-69622 cedex, France

## Abstract

**Background:**

Expressed sequence tag (EST) databases represent a valuable resource for the identification of genes in organisms with uncharacterized genomes and for development of molecular markers. One class of markers derived from EST sequences are simple sequence repeat (SSR) markers, also known as EST-SSRs. These are useful in plant genetic and evolutionary studies because they are located in transcribed genes and a putative function can often be inferred from homology searches. Another important feature of EST-SSR markers is their expected high level of transferability to related species that makes them very promising for comparative mapping. In the present study we constructed a normalized EST library from floral tissue of *Silene latifolia *with the aim to identify expressed genes and to develop polymorphic molecular markers.

**Results:**

We obtained a total of 3662 high quality sequences from a normalized *Silene *cDNA library. These represent 3105 unigenes, with 73% of unigenes matching genes in other species. We found 255 sequences containing one or more SSR motifs. More than 60% of these SSRs were trinucleotides. A total of 30 microsatellite loci were identified from 106 ESTs having sufficient flanking sequences for primer design. The inheritance of these loci was tested via segregation analyses and their usefulness for linkage mapping was assessed in an interspecific cross. Tests for crossamplification of the EST-SSR loci in other *Silene *species established their applicability to related species.

**Conclusion:**

The newly characterized genes and gene-derived markers from our *Silene *EST library represent a valuable genetic resource for future studies on *Silene latifolia *and related species. The polymorphism and transferability of EST-SSR markers facilitate comparative linkage mapping and analyses of genetic diversity in the genus *Silene*.

## Background

The White Campion, *Silene latifolia *Poiret, a member of the plant family Caryophyllaceae, is a dioecious herb. The species is diploid, has a large nuclear genome size (1C = 2646 Mbp [1]) and a haploid chromosome number of 12. Sex is determined genetically by heteromorphic sex chromosomes that were first described by Blackburn [2] and Winge [3]. As in humans, females are homogametic, XX, and males are the heterogametic sex, XY. The sex chromosomes are the largest chromosomes and contribute substantially to the large genome size of this species. Although dioecy has evolved many times in different plant lineages [4], well differentiated, heteromorphic sex chromosomes are relatively rare in plants. Over the last decades, *Silene latifolia *has become a model organism in plant ecology and evolution. Major research avenues include for example the evolution of heteromorphic sex chromosomes in plants [5-7], sexual dimorphism [8,9], plant-pathogen [10] and plant pollinator interactions [11], invasive plant biology [12], hybridization and introgression [13], and habitat adaptation [14].

To address these ecological and evolutionary questions, a diverse set of molecular markers has been used to date in *Silene latifolia*. Only recently have the first genomic simple sequence repeats (SSRs), also known as microsatellites, been identified and used [15]. Current limitations of the available markers include the problems that the widely used AFLPs, and formerly RAPDs, are anonymous and dominant markers. While AFLP markers have been used successfully for linkage mapping in the related *Silene vulgaris*, the resulting maps derived from the maternal and paternal parent, respectively, could not be joined into a single, unifying map, because of the limited information on coupling phase provided by dominant markers [16]. Similarly, a recent genome scan analysis for markers under selection identified several AFLP markers that carried the signature of selection [17], but characterization of the outlier markers failed to identify transcribed genes. To overcome such limitations, we have embarked on an expressed sequence tag (EST) project to identify transcribed genes in *S. latifolia *and to characterize simple sequence repeats in these expressed genes. SSR markers identified in EST sequences are known as EST-SSRs.

SSRs are tandemly repeated tracts of DNA composed of 1–6 base pair (bp) long units. They are ubiquitous in prokaryotes and eukaryotes [18], both in coding and noncoding regions, and are usually characterized by a high degree of length polymorphism. SSR markers are useful for a variety of applications, because of their multiallelic nature, codominant inheritance, relative abundance, and good genome coverage. The conservation of flanking sequences in the vicinity of the repeat motifs often permits the genotyping of related species with a single primer set [19]. Microsatellites have proven to be an extremely valuable tool for genome mapping in many organisms [20,21], but their applications span over different areas ranging from ancient and forensic DNA studies to population genetics and conservation/management of biological resources [22]. Moreover, microsatellites, due to their large amount of variability, have the potential to be informative about gene and genome duplication, but only recently have been used for these topics [23].

Expressed sequence tags (ESTs) are sequenced portions of messenger RNA. In recent years, EST projects have been initiated for numerous plant and animal species, and have generated a vast amount of sequence information that can be used for gene discovery, functional genetic studies, and marker development [24]. SSRs are relatively common in expressed genes, mainly in the 5' and 3' untranslated regions (UTRs). Such EST-SSRs, or genic SSRs, have several advantages compared to other molecular markers. First, studies in plants, animals, and fungi have shown that EST-SSRs are often more widely transferable between species, and even genera, than genomic SSR [25,26]. Second, because they are located in transcribed genes, the identification of outlier EST-SSRs loci in genome scan analyses may directly identify candidates for genes under selection [27,28]. By comparing the EST sequence to protein sequence databases, it may further be possible to shed light on the functional identity of the gene. Third, the increased likelihood for cross-species amplification and the codominant nature of EST-SSRs make them ideal markers for comparative mapping [29,30]. Fourth, EST-SSRs often display reduced levels of polymorphism compared to genomic SSRs [31,32], which may facilitate genotyping and allow a more accurate estimates of allele frequencies in population genetic studies compare to hypervariable loci. Finally, the ease with which EST-SSRs can be mapped may facilitate the identification of new genes that are linked to traits of particular interest, such as to the sex chromosomes in *S. latifolia*.

As codominant markers, EST-SSRs are expected to segregate according to Mendel's laws in crosses between individuals, and Mendelian inheritance of alleles is a requirement for population genetic analyses. An earlier review of microsatellite inheritance studies found that Mendelian inheritance was almost never rejected for diploid vertebrate species [22,33]. However, there is increasing evidence of what appear to be "non-Mendelian" patterns of inheritance of microsatellites [34,35]. Because relatively few studies report tests for Mendelian inheritance, it is still unclear how common non-Mendelian inheritance is. A large fraction of "non-Mendelian" ratios of alleles in offspring of experimental crosses is apparently caused by null alleles [36]. Potential causes of non-Mendelian behaviour include sex linkage, physical association with genes under strong selection, transposable elements, or processes such as non-disjunction or meiotic drive that act during meiosis [36,37]. To use EST-SSR loci for population genetics, it is thus essential that Mendelian segregation be verified in controlled crosses.

We constructed an EST library from floral tissue of male and female *S. latifolia *with the aim to identify expressed genes and to develop polymorphic molecular markers. Floral tissue was chosen because we are interested in the genetic basis of sex determination in dioecious *Silene*, the evolution of plant sex chromosomes, and floral isolation between *S. latifolia *and the closely related *S. dioica*.

In this paper we first describe our EST library and show that this library is a rich source of genes that may be involved in flower development and the control of floral trait differences between male and female plants, but also between *S. latifolia *and related species. Second, we describe a set of newly characterized EST-SSR loci and provide information about their polymorphism, Mendelian inheritance, and transferability to other *Silene *species. Finally, we report on the utility of these markers for comparative mapping.

## Results

### EST library characterization

Random 5' sequencing of our directional cDNA library resulted in 3662 high quality sequences with an average length of 609 nucleotides. Assembly using TGICL resulted in 3105 unigenes, consisting of 2673 singlets and 432 contigs. Average unigene length (625.5 bp) was shorter than average contig length (682.6 bp). Most contigs (80.6%) contained 2 ESTs. Only 25 contigs (5.8%) contained 4 or more sequences and the largest number of sequences per contig was 7 (Table 1).

**Table 1:** *Silene latifolia *EST library and sequencing statistics

Library titer (cfu/μl)	2.9 × 10^3^
Total number recombinant clones	7.5 × 10^6^
Average cDNA insert size	1000 bp
Average good sequence length	609 bp
Total sequences	4416
Sequences passed quality check	3662 (83%)
Number of singlets	2673
Number of contigs	432
Unique gene sequences (unigenes)	3105
Average unigene length	625.5 bp
Observed redundancy *	17.9%

* (EST # after quality check – unigene #)/unigene # [39]

During pre-processing, all sequences were searched for the tags identifying three different pools. Of the 3662 high quality sequences, 1342 were from pool A (petals of male and female flowers), 1385 from pool B (male buds and flowers), and 843 from pool C (female buds and flowers). In 92 sequences (2.5%) the adaptor could not be retrieved. Despite the small number of ESTs per contig, most contigs (76%) consisting of four or more ESTs contained sequences from more than one tissue pool. Only 6 such contigs were made up by sequences from a single tissue pool. Of these, 4 contigs combined sequences from pool B, and one contig each combined sequences from pools A and C.

### EST annotation and functional classification

We used BLASTX to annotate our *Silene latifolia *unigene sequences. 2271 (73%) of the unigenes matched genes in other species with an expectation value of 1e-10 or better in a search against the NCBI nr protein database (release June 2008). Best hits in BLASTX searches were mainly to *Vitis vinifera *(940 hits, 30.3% of all unigenes), *Arabidopsis thaliana *(317 hits, 10.2%), *Populus trichocarpa *(238 hits, 7.7%), and *Oryza sativa *(98 hits, 3.2%). Out of our 3105 unigenes, only twenty-six had a best hit with a *Silene *species (0.8%). Several unigenes identified in our EST library have been identified as putative homologs of *Arabidopsis *genes that are implicated in floral development (Table 2).

**Table 2:** *Silene latifolia *unigenes tagged with GO term flower development and best BLASTX hits to *Arabidopsis *genes

Annotation	Ath Gene ID	*E*-value/Identities
Serine/threonine protein phosphatase	At3G19980	9e-178/95%
Copper chaperone	At3G56240	2e-27/65%
MYB domain protein 21	At3G27810	2e-58/89%
Ribosomal protein L24	At2G36620	5e-59/90%
Zinc finger family protein	At3G09320	3e-53/73%
ROOT MERISTEMLESS 1/CADMIUM	At4G23100	7e-58/81%
SENSITIVE 2		
EARLY FLOWERING 4	At2G40080	3e-22/61%
SEPALLATA2	At3G02310	4e-68/84%
Stress enhanced protein 2	At2G21970	9e-31/50%
Alpha-galactosidase 1	At5G08380	3e-74/80%

**Figure 1:**
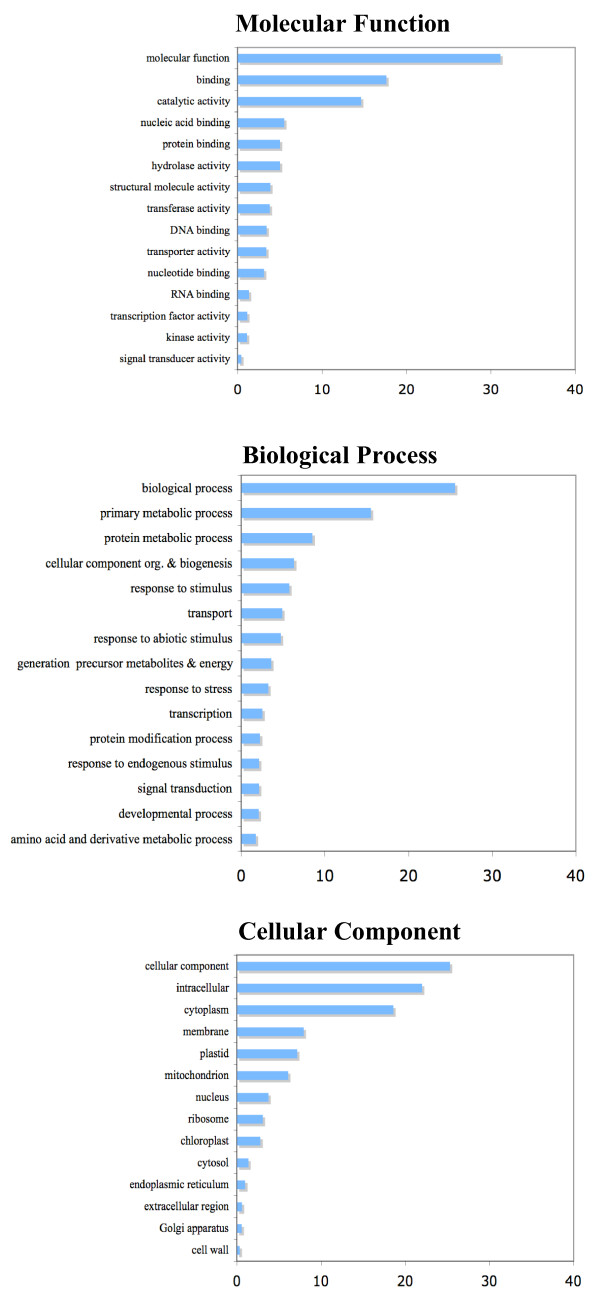
**Gene Ontology (GO) classification of the *Silene latifolia *EST library**. The relative frequencies of GO hits for *Silene latifolia *unigenes assigned to the GO functional categories Biological Process, Molecular Function, and Cellular Component, as defined for the *Arabidopsis *proteome.

Gene Ontology (GO) [38] annotation was performed with BLAST2GO. In total, 1837 unigenes were annotated with 7215 GO terms. At least one Biological Process was proposed for 1308 unigenes, a Cellular Component for 1404 unigenes, and 1306 unigenes were annotated with at least one Molecular Function. There were 863 sequences with annotations for all three GO categories (Biological Process, Cellular Component and Molecular Function) and 1318 unigenes had annotations for at least 2 categories. The relative frequencies of GO hits are shown in Fig. 1.

### Characterization of microsatellite motifs

We identified 255 sequences containing one or more microsatellite motifs in the library screen. The observed frequencies of di-, tri-, tetra-, penta- and hexa-repeats were 16.8% (43), 64.3% (164), 15.6% (40), 5.4% (14) and 5.4% (14), respectively. The 43 di-nucleotide repeat sequences consisted of (TC)/(GA)_n_, (AT)/(TA)_n _and (TG)/(CA)_n_. Among the di-nucleotide repeats there was a distinct predominance of (TC)/(GA)_n _repeats (72%, 31/43), with low frequencies of other di-nucleotide repeats, (AT)_n _and (TG)_n _(16.2%, 7/43 and 11.6%, 5/43 respectively). 164 tri-nucleotide repeat motifs were recognized, which represents the most frequent repeat unit (64.3%, 164/255). Their motifs included (ATA)/(TAT)_n_, (AGC)/(GCT)_n_, (AGA)/(TCT)_n_, (AAC)/(GTT)_n_, (ATC)/(GAT)_n_, (CCA)/(TGG)_n_, (GTA)/(TAC)_n_, (GGA)/(TCC)_n_, (CGC)/(GCG)_n_, and (GAC)/(GTC)_n_. Of these, the motif (ATA)/(TAT)_n _was the most frequent (29.2%, 48/164), followed by (AGA)/(TCT)_n _(23.1%, 38/164), (ATC)/(GAT)_n _(17.6%, 29/164), (AAC)/(GTT)_n _(10.3%, 17/164), and (GGA)/(TCC)_n _(6.7%, 11/164). (AGC)/(GCT)_n_, (CCA)/(TGG)_n_, (GTA)/(TAC)_n_, (CGC)/(GCG) and (GAC)/(GTC) showed a very low frequency. Tetra-nt repeat motifs were identified in 40 different clones (15.6%); these included (ATTT)/(AAAT)_n_, (ATCA)/(TGAT)_n_, (AATT)/(AATT)_n_, (AAGA)/(TCTT)_n_, (AACC)/(GGTT)_n_, (ATGA)/(TCAT)_n_, (CAAA)/(TTTG)_n_, (TATC)/(GATA)_n_, (GGAG)/(CTCC)_n_, (TAGT)/(ACTA)_n_, (CTTC)/(GAAG)_n_, (TAGC)/(GCTA)_n _and (TCAC)/(GTGA)_n_. The most frequent 4-nt repeat motifs were (CAAA)/(TTTG)_n_, (ATTT)/(AAAT)_n_, (ATCA)/(TGAT)_n_, (AATT)/(AATT)_n_, but their frequency was low (12.4%, 5/40). We identified 5 and 6 different penta or hexa-nt SSR motifs, most of them were found only once.

### Identification of polymorphic markers

Among the 255 SSR-containing unigenes we selected 106 that had long enough sequences flanking the SSR to design primer pairs. 74 primer pairs that were not likely to form internal secondary structures were designed and tested by amplifying template DNA from *S. latifolia*. After some optimization, 61 of these primer pairs were successfully amplified. The other 13 primer pairs failed. Among the working primer pairs, 49 produced PCR products of the expected size, 10 produced PCR fragments that were considerably longer than expected and the rest produced multiple bands. Finally, about 66% of the primers that were initially designed appeared to amplify the expected product as judged from agarose-gel electrophoresis. However, when these PCR products were subsequently analyzed on a capillary sequencer, some of them were not scorable due to excessive "stutter bands". Finally, 30 primer pairs remained (Additional file 1).

In order to obtain information on the putative identities and functions of the genes containing EST-SSRs, the corresponding unigene sequences were subjected to BLASTX searches against the *Arabidopsis thaliana *Refseq database. About 90% of EST-SSRs matched *Arabidopsis *genes with an expectation value of 1e-10 or better (see Additional file 2). The position of the SSR motifs was unambiguously identified for 24 out of the 30 loci. For five loci, the two methods used to infer the SSR position disagreed and for one locus, no similarity was found to known *Arabidopsis *genes. Of the 24 loci, 11 are located in protein coding sequences (CDS), 11 are located in the 5' UTR, and 2 in the 3' UTR. The level of polymorphism as estimated from the polymorphic information content (PIC) was higher in loci located in untranslated regions (5' and 3' UTRs) than in loci located in coding regions (PIC = 0.656 vs PIC = 0.543, respectively).

### EST-SSR Polymorphism

We surveyed the allelic variability of the markers by genotyping individuals from a natural population of *S. latifolia*. Most microsatellite loci showed allelic polymorphism. The number of alleles per locus varied from 2 to 12 in the panel of 30 individuals, and the PIC values ranged from 0.27 to 0.81 with a mean value of 0.55. The mean number of alleles per locus was 6.65 alleles, the mean observed heterozygosity was 0.50, and the mean expected heretozygosity was 0.63 (see Additional file 1). A small proportion of the microsatellite markers (13.7%) amplified more than two alleles in some individuals, indicating that these primer pairs may be amplifying duplicated loci. Duplicated loci can share alleles of the same length so that alleles cannot unambiguously be assigned to one locus or the other. Therefore we were hesitant to use these loci for population genetic analysis. However, it is possible to use these duplicated loci in other applications such as gene mapping or the study of gene duplication. Additional file 1 lists the repeat unit found in the original EST sequence, together with the primer sequences that were used to PCR amplify the microsatellite loci; additionally, allele size ranges, genomic position of SSR and the number of alleles observed among the samples studied are given for each locus. Exact tests for Hardy-Weinberg equilibrium revealed that the majority of these microsatellites were in HWE, but 6 loci (Locus SL_eSSR02, Locus SL_eSSR06, Locus SL_eSSR11, Locus SL_eSSR16, Locus SL_eSSR17 and Locus SL_eSSR24) showed significant departure from HWE (p < 0.002) after Bonferroni correction (see Additional file 1). Of these, 5 loci revealed a heterozygote deficit and one a heterozygote excess.

### Segregation analysis

Of the 30 EST-SSR loci developed in this study, 25 were polymorphic in an experimental interspecific cross between *S. latifolia *and *S. dioica*. These loci were tested for Mendelian segregation in 90 progeny. Null alleles were deduced at some loci where unexpected progeny genotypes could be explained only by null alleles in the parents. As indicated by the X^2 ^contingency test, most SSRs segregated in Mendelian ratios, but 7 loci showed significant segregation distortion after Bonferroni correction for multiple testing (Table 3).

**Table 3:** Segregation analysis of EST-SSR markers in a *Silene *cross.

Locus	Male × female	Genotype of progeny	Expected ratio	Observed ratio	X^2^
SL_eSSR01	A/B × A/B	A/A:A/B:B/B	1:2:1	60:5:17	108.31*
SL_eSSR02	A/B × A/B	A/A:A/B:B/B	1:2:1	17:33:33	9.65
SL_eSSR03	A/B^a ^× A/C	A/A:A/C:A/B:B/C	1:1:1:1	18:8:34:13	19.35*
SL_eSSR04	A/B × A/B	A/A:A/B:B/B	1:2:1	27:52:2	21.96*
SL_eSSR05	A/B × C/D	A/C:A/D:B/C:B/D	1:1:1:1	21:5:25:29	16.60*
SL_eSSR06	A/B × A/C	A/A:A/C:A/B:B/C	1:1:1:1	8:25:21:27	8.55
SL_eSSR07	A/B × A/B	A/A:A/B:B/B	1:2:1	17:43:21	0.70
SL_eSSR08	A/B × A/B	A/A:A/B:B/B	1:2:1	22:23:32	15.07*
SL_eSSR09	A/B × A/B	A/A:A/B:B/B	1:2:1	4:32:19	9.65
SL_eSSR10	A/B × A/B	A/A:A/B:B/B	1:2:1	19:43:21	0.20
SL_eSSR11	A/B × C/D^a^	A/C:A/D:B/C:B/D	1:1:1:1	21:13:28:21	5.43
SL_eSSR12	A/B × A/C	A/A:A/C:A/B:B/C	1:1:1:1	27:16:21:19	3.12
SL_eSSR13	A/B × A/C	A/A:A/C:A/B:B/C	1:1:1:1	26:13:22:18	4.70
SL_eSSR14	A/B × A/B	A/A:A/B:B/B	1:2:1	24:43:19	0.58
SL_eSSR16	A/B × A/B	A/A:A/B:B/B	1:2:1	16:45:17	1.87
SL_eSSR17	A/B × A/B	A/A:A/B:B/B	1:2:1	8:53:18	11.92
SL_eSSR20	A/B × A/B	A/A:A/B:B/B	1:2:1	15:28:15	0.06
SL_eSSR21	A/B × A/A	A/B × A/A	1:1	57:22	15.50*
SL_eSSR22	A/B × A/C	A/A:A/C:A/B:B/C	1:1:1:1	5:24:19:23	13.00
SL_eSSR24	A/B × A/A	A/B × A/A	1:1	47:11	24.27*
SL_eSSR25	A/B × C/D	A/C:A/D:B/C:B/D	1:1:1:1	21:19:20:23	0.42
SL_eSSR26	A/B × A/B	A/A:A/B:B/B	1:2:1	31:36:19	0.31
SL_eSSR27	A/B × A/A	A/B × A/A	1:1	41:21	6.45
SL_eSSR28	A/B × A/C	A/A:A/C:A/B:B/C	1:1:1:1	22:21:21:13	2.74
SL_eSSR29	A/B × A/C	A/A:A/C:A/B:B/C	1:1:1:1	22:23:21:18	0.66

*Significant deviation from expected Mendelian ratio after Bonferroni correction (P < 0.002)

^a ^indicates an inferred null allele

90 F2 offspring from a cross between *Silene latifolia *and *S. dioica *were genotyped and segregation ratios tested.

### Transferability of EST-SSR loci

Among the 30 microsatellite primers tested for amplification in other *Silene *species, 93% amplified a product of expected size in *S. dioica*, 90% in *S. diclinis*, 63% in *S. nutans*, 57% in *S. acaulis *and 47% of the primer pairs were transferable to *S. vulgaris, S. colpophylla *and *S. ciliata *(Table 4).

**Table 4:** Cross-species amplification of *S. latifolia *EST-SSRs

Locus	size range (bp)						
	*S. dioica*	*S. diclinis*	*S. vulgaris*	*S. nutans*	*S. acaulis*	*S. colpophylla*	*S. ciliata*
	
SL_eSSR01	223–236	226	218–230	227	390	-	-
SL_eSSR02	209–228	217–221	-	182–235	271–273	213	230
SL_eSSR03	210–262^+^	241	236–245	-	-	-	-
SL_eSSR04	164–184	160–175	160–164	167–170	170	173–182	-
SL_eSSR05	233–240	235–249	184–198	236–239	219–239	235–258^+^	218–220
SL_eSSR06	156–176	173–185	174–183	-	-	-	-
SL_eSSR07	164–178	173–183	-	-	181	-	-
SL_eSSR08	235–270	252–261	-	222–225	214–237	227–237	230–248
SL_eSSR09	234	234	-	-	-	-	-
SL_eSSR10	332–356	351–362	-	-	-	-	-
SL_eSSR11	-	-	-	-	-	-	-
SL_eSSR12	170–187	173–179	174–183	191	186–196	199–201	173–177^+^
SL_eSSR13	206–216	214	-	208–286	208–286	-	208–286
SL_eSSR14	326–329	338	-	160	-	-	338–400
SL_eSSR15	219–236	257–281^+^	-	-	-	-	-
SL_eSSR16	185–198	192–200	161–169	175–183	192–206	178	197–204
SL_eSSR17	231–289	242–246	209–223	222–230	227	228–230	195
SL_eSSR18	267–291	250–252	-	277	257–263	-	-
SL_eSSR19	182–198	-	-	190	190–205	-	180
SL_eSSR20	194–200	202	167–207	187–196	202	202	205
SL_eSSR21	236–241	223	-	-	-	239	-
SL_eSSR22	166–190	172–184	166–170	185	160–166	167–185^+^	151–184
SL_eSSR23	211–217	202–208	-	187	187	178–187	-
SL_eSSR24	198–212	210	-	-	-	-	-
SL_eSSR25	227–262^+^	227–253	201–262^+^	-	-	201–245	241
SL_eSSR26	207–213	210	208	210	-	214–223	211
SL_eSSR27	236–251	245–257	239–248	245	243–255	239–248^+^	245
SL_eSSR28	190–210	195	195–212	204	192–204	-	-
SL_eSSR29	214–233	232–247	-	236–247	-	-	-
SL_eSSR30	-	-	-	-	-	-	-

- no amplification

^+ ^duplicated locus

### Utility of EST-SSRs for mapping

Linkage mapping based on 25 markers polymorphic in an interspecific cross between *S. latifolia *and *S. dioica *led to the identification of 6 linkage groups when a LOD value of 3.0 was employed. These 6 linkage groups encompassed 17 EST-SSRs. 8 markers remained unlinked. These unlinked markers are located on other linkage groups that harbour only one or few weakly linked EST-SSRs, as indicated by the fact that in combination with dominant AFLP markers, all EST-SSR markers map to one of 12 linkage groups (unpublished results), which corresponds to the haploid chromosome number in dioecious *Silene*.

## Discussion

The White Campion, *Silene latifolia*, has long been used as a model organism for a wide range of ecological and evolutionary questions. Despite this great interest in the species, no systematic attempts have been made to characterize large numbers of genes across the species' genome and to identify gene-specific markers that can be used for a wide range of research questions and can also be transferred to closely related species. Our EST library and the molecular markers derived from this library may therefore provide a valuable molecular tool for further studies on this ecological model organism.

### EST library annotation

Our normalized floral cDNA library displayed a low observed redundancy and was efficient to identify a large number of previously uncharacterized genes in *S. latifolia *that represent all major categories in the Gene Ontology (GO) classification. This confirms that even a limited EST dataset represents a valuable resource for molecular non-model organisms [39]. The great majority of unigenes identified in the present study (73%) had a significant similarity with genes in other plant species. Most similarities were found to *Vitis vinifera*, *Arabidopsis thaliana*, and *Populus trichocarpa*. These species are all core eudicots and belong to the rosids, whereas *Silene *belongs to the core eudicot clade Caryophyllales [40]. The fact that similarities were most often found to these particular plant species is a consequence of the fact that all these species have fully sequenced genomes and large EST databases, and does not reflect their phylogenetic proximity to *Silene*. Similarities with genes from these species provided some insights into the identities of *Silene *genes. However, BLAST-based annotations, especially of short EST sequences, can be misleading when hits are due to domain homologies, rather than homology to orthologs [39].

The few hits (0.8%) to *Silene *sequences available in GenBank reflect the lack of sequence information for this genus and emphasizes the value of the EST dataset developed in the present study.

The great majority of contigs with of more than four EST reads combined sequences derived from more than one tissue. Of the 6 contigs for which all ESTs were derived from a single tissue, four contained sequences expressed in male flower buds (pool B). Two of these contigs had strong similarities with genes that were previously found to be expressed exclusively in males. One contig had high similarity (8e-12) to MROS4 [41] and another one was similar (7e-12) to Men-1 [42]. To what extend the other genes that were found to be expressed in either males (pool B, 2 more contigs) or females (pool C, 1 contig) in the present study are indeed sex-specifically expressed remains to be tested experimentally.

### Floral development genes

We identified several unigenes that are putative homologs of *Arabidopsis *genes that are involved in floral development (Table 2). These include genes that perceive or respond to environmental signals such as *EARLY FLOWERING 4 *(*ELF4*), and stress enhanced protein 2 (Sep2), and transcription factors that control floral development such as SEPALLATA 2 (SEP2), MYB domain protein 21 (MYB21), and zinc finger family proteins.

The transition to flowering in plants is regulated by environmental factors such as temperature and light. Day-length sensing involves an interaction between the relative length of day and night, and endogenous rhythms that are controlled by the plant circadian clock. The gene *EARLY FLOWERING 4 *(*ELF4*) is involved in photoperiod perception and circadian regulation [43]. The expression of stress enhanced protein 2 is induced specifically by light stress and is specific, because other physiological stresses such as cold, heat, or salt do not promote accumulation of Sep2 transcripts [44].

SEPALLATA (SEP) genes form a subfamily of MADS-box transcription factors that are critical for a number of developmental processes. In particular, the SEPALLATA (SEP) genes play an important role in controlling the development of floral organs in flowering plants. In *Arabidopsis thaliana*, SEP1, SEP 2, SEP3 and SEP4 are required for specifying the identity of all four whorls of floral organs, and for the floral meristem determination [45,46]. MYB proteins are transcription factors that are characterize by a MYB (DNA-binding) domain. MYB21 is specifically expressed in flowers in *A. thaliana *and directly activates the expression of genes involved in the phenylpropanoid metabolism [47].

Analysis of expression patterns of putative homologs of these *Arabidopsis *genes in *S. latifolia *will reveal to what extend their functions are conserved in *Silene *and may help to elucidate their roles in *Silene *flower development.

### SSR Frequency and distribution

In this study we found trinucleotide repeats (TNRs) to be the most common SSR type in ESTs of *Silene latifolia*. This is in agreement with a majority of studies that report TNRs as the most abundant class of SSRs in plant ESTs [48,49], in contrast to recent studies in *Actinidia *[50] and *Picea *species [51] wherein dinucleotide repeats (DNRs) were found to be the most abundant class of EST-SSRs. Interestingly, DNRs have been reported to be the most abundant SSRs in ESTs of many animal species such as medaka, *Fundulus*, zebrafish, and *Xiphophorus *[52].

Among the trimeric motifs, (ATA)_n_, (AGA)_n _and (ATC)_n _were the most common (70%) in *S. latifolia*. In rice, 60% of EST-derived microsatellite sequences were (CCG)_n_, (ACG)_n_, (AGG)_n _and (ACC)_n _[53], and in maize (CCG)_n _and (AGG)_n _were most abundant [54]. (CCG)_n _was also the most common motif in sugarcane [55]. In contrast, the motifs (ATC)_n _and (AAG)_n _represented 60% of all microsatellite motifs of the dicotyledon *Arabidopsis *[56]. The motif (AAT)_n _was found to be rare in barley, rice, maize and sugarcane, as well as in *Arabidopsis*, and was not found here in *S. latifolia*. A possible reason for its rarity is that TAA-based variants code for stop codons have a direct effect on protein synthesis [54]. Of the dimeric repeats, the motif (TC)_n _was the most common in our dataset with 76% of dinucleotide repeats, whereas no (CG)_n _motif was found.

In plants, TC and CTT repeats (referred to as AGA in this study) were found to be typical of transcribed regions and to occur with high frequency in the 5' UTRs. It has also been suggested that the high level of the (TC)_n _motif is due to its translation into Ala and Leu, depending on the reading frame [57]; Ala and Leu are present in proteins at high frequencies of 8% and 10%, respectively. (AT)_n _repeats have been reported to be very abundant in the genomic sequences of plants [58], but they were relatively rare (16%) in our *Silene *EST sequences. The deficiency of AT-SSRs in our EST sequences is in accordance with reports from rice [53], *Arabidopsis *[56] and maize [54]. Overall, GC-rich SSR motifs were less frequent in *Silene *ESTs than GC-poor motifs. This was most evident in the relative abundance of (GA)/(AGA)_n _and deficiency of (CG)/(CCG)_n _repeat motifs among the DNARs/TNRs, respectively, identified in this study. Interestingly, a similar difference in SSR motif in ESTs has been reported earlier, and seems to be a common feature of the dicotyledon species [56,59].

### EST-SSR marker polymorphism

The majority of *S. latifolia *EST-SSRs generated high-quality amplification products, suggesting that ESTs are ideally suited for specific primer design. In this study, PCR amplification was successful for 82% of the primer pairs designed from ESTs. Among the primer pairs that amplified, we noticed that in some cases the amplification product was substantially larger than expected from the EST sequence analysis. This increase in product size was most likely due to the presence of introns and large insertions in the corresponding genomic sequence. Other primer pairs (18%) failed to amplify a PCR product. Generally, inconsistent amplification or amplification failure of EST-SSR loci may arise as a result of factors such as the presence of introns that are too large for efficient amplification, the use of poor quality sequences for primer design, and mutational substitutions, insertions or deletions within the priming site [60].

Levels of polymorphism detected with EST-SSRs have been compared in several studies to those revealed by genomic SSRs. In most cases, the latter were found to be more polymorphic [61]. Our EST-SSRs revealed relatively low levels of polymorphism in the *S. latifolia *population surveyed as indicated by the average number of alleles per locus (6.65) and the average He (0.63) and Ho (0.50). A study based on genomic SSRs [15] detected substantially higher levels of polymorphism in SSRs, with 25–43 alleles per locus and He between 0.86 to 0.97 and Ho between 0.23 to 1.0.

All polymorphic loci developed in the present study were tested for deviations from Hardy-Weinberg equilibrium (HWE). We observed significant deviations from HWE at six loci (20%) after Bonferroni correction in a natural *S. latifolia *population. The fact that only a minority of surveyed loci revealed deviations from HWE indicates that the investigated population overall is in Hardy-Weinberg equilibrium, and that deviations at individual loci are most likely due to locus-specific effects, and not due to biological factors such as inbreeding or genetic drift, which would affect all loci.

Null alleles are known to be a major cause of heterozygote deficiencies observed in SSR analyses of animal and plant populations [62]. Null alleles most commonly arise from point mutations in the sequence flanking the repeat region [63], which reduces or prevents primer annealing. Null alleles can also be generated via differential amplification of size-variant alleles [64]. Due to the competitive nature of the PCR process, shorter alleles often amplify more efficiently than larger ones, such that only the smaller of two alleles may be detected from a heterozygous individual. In general, null alleles complicate the interpretation of microsatellite data because of the reduced level of observed heterozygosity [62]. Problems with null alleles can be ameliorated by improvements in primer design [37]. In addition to these technical problems, several population genetic phenomena may give the false impression that microsatellite null alleles are present in a given study. Biological factors such as Wahlund effect or inbreeding, for example, can cause significant heterozygote deficits relative to HWE that might be misconstrued as evidence for null alleles [65]. The use of a large number of SSR loci may help to distinguish between locus-specific problems and biological processes leading to heterozygote deficiencies.

A further potential cause of deviations from Hardy-Weinberg expectations involves sex-linkage. The divergence between the X and Y chromosomes in species with heterogametic males (or of W and Z chromosomes in species with heterogametic females) often leads to the phenomenon that only one allele is amplified in the heterogametic sex, although sex chromosomes typically evolved from ancestral autosomes. Thus, if sex-linkage remains unrecognized at a locus, an associated locus-specific heterozygote deficit may be wrongly interpreted as indicative of null alleles. Indeed, one locus that maps to linkage group 1, which corresponds to the sex chromosome, reveals significant deviation from HWE after Bonferroni correction.

### Mendelian segregation of EST-SSR markers

We used plants from an experimental cross to assess Mendelian inheritance of our EST-SSR loci. 28% of the loci that were polymorphic in the cross showed a significant deviation from expected Mendelian ratios after Bonferrroni correction (p < 0.002). Segregation distortion may be caused by technical problems, including null alleles, but may also have a biological basis, such as gametic selection, embryogenesis, seed set or sex linkage. In addition, segregation distortion may occur as a consequence of the divergence between the two species. Although markers displaying segregation distortion may complicate linkage analysis [66], distorted loci can often be mapped, and the mapping of distorted markers may help to identify genes that have important biological functions [67,68].

### Transferability of EST-SSR markers

By virtue of the sequence conservation of transcribed regions of the genome, a significant portion of the primer pairs designed from EST-SSRs is expected to function in distantly related species. Transferability of EST-derived markers over different taxonomic levels has been demonstrated earlier [55,69]. In our study, the majority of our 30 EST-derived SSR loci from *S. latifolia *revealed cross-species amplification with alleles of comparable sizes in one or several of the tested *Silene *species. As expected, the transferability of the markers was higher for *S. dioica *and *S. diclinis *than for the other species, because of their close phylogenetic relatedness to *S. latifolia*. High amplification success suggests that the flanking regions of these loci are sufficiently conserved, and that these loci can be used for comparative analyses of genetic diversity in the genus *Silene*. In addition, these genic SSRs are good candidates for the development of conserved orthologous markers for linkage mapping and QTL analyses in different *Silene *species [16,70].

### EST-SSR markers for comparative mapping

The abundance of microsatellites in transcribed regions of the genome and the level of polymorphism of these markers make EST libraries a valuable source of markers for genetic mapping. The high proportion of informative markers (83%) found in the present study for an interspecific cross between two closely related dioecious *Silene *species, and the identification of 6 out of 12 expected linkage groups, including the sex chromosome, reveal that these EST-SSR loci are valuable markers for linkage mapping in *S. latifolia *and related dioecious species.

Perhaps the most important feature of the EST-SSR markers for comparative linkage mapping is that they are transferable also to more distantly related species. The value of the transferability of such markers to related species for the purpose of comparative mapping has been demonstrated in several studies in wheat, rye and rice [30,71]. Our study shows that 93 to 47% of EST-SSR primer pairs designed for *S. latifolia *will also yield amplicons in *S. dioica, S. diclinis, S. vulgaris, S. colpophylla *and *S. ciliata *and thus provide valuable markers for comparative linkage mapping in these species. With these markers, new insights can, for example, be gained into the independent evolution of sex chromosomes from ancestral autosomes in the genus *Silene*, a topic that has recently received great interest [72,73]. Moreover, the EST-SSR loci that were found to map to the *S. latifolia *sex chromosomes represent a set of newly identified sex-linked genes that are now being used to further explore the divergence between the X and Y chromosomes in this species.

## Conclusion

Only few microsatellite markers have to date been described in the literature for *Silene *species [15,74] and all of these are genomic SSRs for which no information on transferability to other species is available. Thus, the set of 30 EST-SSR markers reported in this study represents an important resource for future studies on *S. latifolia *and other species of this highly diverse genus. Most notably, these EST-SSRs will allow to perform comparative analyses of population structure, help with the identification of loci under selection in population genomic studies, and facilitate comparative linkage mapping in the genus *Silene*.

## Methods

### Library construction and EST isolation

A cDNA library was constructed from polyA^+ ^RNA isolated from flower buds and open flowers of male and female *S. latifolia *grown in a greenhouse at ETH Zurich under long-day light conditions. Plants were grown from seeds collected in a natural population in Switzerland (site Leuk in Valais, Switzerland; 46°19'/7°39'). Floral tissue was collected at 10 pm in the dark. At this time, flowers are fully open and emit a strong scent [10]. Three tissue pools were prepared. Pool A included petals from fully open male and female flowers; pool B consisted of male buds and flowers; pool C contained female buds and flowers. RNA was isolated using TriFast (PeqLab), stored in liquid nitrogen, and sent to GATC Biotech (Konstanz, Germany) for library construction. There, first-strand cDNA synthesis was performed with M-MLV-RNase H^- ^reverse transcriptase and a different oligo (dT)-*Not I *primer for each cDNA pool. Each one of these primers contained at the 3' end a specific 3 bp tag: pool A contained the tag 'TCG', pool B the tag 'GAG', and pool C the tag 'ATG'. Resulting cDNA was amplified with 10 cycles of LA-PCR. To normalize cDNA, one cycle of denaturation and reassociation of the cDNA was performed. Reassociated ds cDNA was separated from the remaining ss cDNA by passing the mixture over a hydroxyapatite column. After hydroxyapatite chromatography, the ss cDNA was amplified with 12 LA-PCR cycles. For directional cloning, the normalized cDNA was first subjected to a limited exonuclease treatment to generate *Eco RI *overhangs at the 5' ends and was then cleaved with *Not I*. Prior to cloning, the cDNA was size fractionated. For this purpose, the cDNA was separated on a 1.3% agarose gel. Following elution of cDNAs larger than 0.5 kb, the cDNA was ligated into *Eco RI *and *Not I *cleaved pBS II SK (+) vector. Ligations were electroporated into Phage T1 resistant TransforMax™ EC100™ (Epicentre) electro-competent cells. After transformation, glycerol was added to a final concentration of 15% (v/v) and the cells were frozen at -70°C in aliquots. After a freezing thawing cycle, the titer of the library was determined to be about 2900 cfu per μl bacterial suspension, which corresponds to about 7.5 × 10^6 ^recombinant clones.

The library was plated out on LB agar plates with Xgal blue/white screening and 0.1% ampicillin, and grown overnight. Positive colonies were picked and grown overnight in 1.5 mL medium with 100 μg/mL ampicillin. The colony stock was then divided in three parts: 200 μl were divided in two plates and archived in LB broth with 15% glycerol at -80°C; the remainder was used for plasmid DNA isolation by using an automated system (BioRobot 3000, Qiagen) and a DirectPrep96 BioRobot kit (Qiagen). Sequence reactions were performed on the plasmid templates by using the Big Dye Terminator v3.1 chemistry (Applied Biosystems) and M13 forward and reverse primers. Sequences were run on an ABI PRISM 3130xl Genetic Analyzer (Applied Biosystems).

### EST processing

We performed a total of 4416 sequencing runs. Raw sequences were extracted from the chromatograms using the PHRED software [75]. Vector, adaptors and potential *E. coli *contaminant sequences were removed using SeqClean [76], with an extra-check performed with Cross-Match [77]. Poly-A sequences were detected and trimmed using SeqClean. Low-complexity regions and repetitive elements were masked using RepeatMasker[78]. This preprocessing phase resulted in 3662 clean EST sequences longer than 100 nucleotides. They were assembled into 3105 « unigenes » (432 contigs + 2673 singlets) using the TGICL software [79] run with default parameters. These « unigenes » represents putative different transcripts from *Silene latifolia *floral tissue. All EST sequences have been submitted to GenBank [GenBank: GH291501 to GH295162].

### EST annotation and function

Unigenes were compared to the NCBI nr (non-redundant) protein database (June 2008) using the BLASTX algorithm and NCBI nr nucleotide database using the BLASTN algorithm. The BLAST2GO [80] annotation tool was used to assign most probable GO terms to the contigs and singlets. Prot4EST [81] (without DECODER) was used to predict CDS. The INTERPROSCAN web service at EBI was used to compare those predicted CDS with known protein motifs and domains.

### Identification of EST-SSRs

The EST library was searched for sequences containing SSRs using Tandem Repeat Finder software [82], available at . All 3105 unigenes were analyzed. Sequences containing di- and tri-nucleotides with at least 5 perfect repeat units, and tetra, penta and hexa-nucleotides with 4 perfect repeat units, were selected for marker development. The mononucleotide A/T repeat was not considered, because of the difficulty of distinguishing real microsatellites from polyadenylation products.

### EST-SSR marker development

Primer pairs flanking repeats were designed using PRIMER3 [83] We used the approach of Schuelke [84] to label PCR products with a fluorescently labelled universal primer, in order to reduce costs. Thus, PCR reactions were performed with three primers: one primer of the microsatellite primer pair was designed with a universal M13 tail attached at its 5' end, the second primer was a normal locus-specific reverse primer, and the third primer was a fluorescently labeled M13 primer. PCR amplifications were conducted in 10 μl reaction volumes containing 10 ng of template DNA, 2 mM MgCl_2_, 0.2 mM dNTPs, 0.2 μM fluorescently labeled M13 primer, 0.2 μM reverse primer, 0.05 μM forward primer (with M13 tail) and 0.05 U Promega GoTaq. The polymerase chain reaction cycling profile was 94°C for 5 min; 30 cycles at 94°C for 30 s, 60°C for 45 s, 72°C for 45 s, followed by 8 cycles 94°C for 30 s, 52°C for 45 s, 72°C for 45 s, and a final extension at 72°C for 10 min. Two PCR products with differences in dye and amplicon size were combined and diluted 1:10. One μl of the diluted sample was added to 9.1 μl of loading mixture made up with 9 μl HiDi formamide and 0.1 μl Genescan 500 LIZ internal size standard (Applied Biosystems). Samples were run on automated DNA sequencer ABI PRISM 3130xl Genetic Analyzer (Applied Biosystems). Output files were analyzed using GeneMapper v4.0 (Applied Biosystems).

### Polymorphism, SSR position and segregation analyses

Successfully amplifying loci were tested for polymorphism by genotyping 30 individuals of *S. latifolia *from a natural population in Leuk, Switzerland [17]. This population contains several hundred individuals that grow in field margins and adjacent fallow land and meadows. Seeds of 30 individuals per population were collected along transects. Care was taken to collect seeds from spatially separated plants (at least 1 m apart) to avoid resampling individuals. Seeds from these seed families were grown in a greenhouse in Zurich. One randomly selected individual per seed family was later used for the analysis of EST-SSR polymorphisms. Voucher individuals are deposited in the herbarium Z/ZT at ETH Zurich under the accession number AW3746. The analyses of polymorphism including allele diversity, observed (H_O_) and expected (H_E_) heterozygosities, Fis, as a measure of heterozygote deficiency or excess [85] and the exact test for deviation from Hardy-Weinberg equilibrium (HWE) were performed using Genepop v3.4. [86], available online at . Polymorphic information content (PIC), a measure of allelic diversity at a given locus, was calculated as follows: , where f_i _is the frequency of the ith allele [87]. To determine whether the SSR motifs were located in protein-coding sequence (CDS) or in untranslated regions (5' or 3' UTRs) we used ESTScan2 [88,89]. In addition, we compared the SSR position with the CDS prediction obtained with Prot4EST and compared the results of both approaches.

The segregation of alleles at 30 microsatellite loci was compared with expected Mendelian ratios by a X^2 ^goodness-of-fit analysis. Segregation ratios were calculated for 90 F2 individuals. These F2 plants were the result of a cross between two F1 individuals that were obtained from an interspecific cross between *S. latifolia *and *S. dioica*. The *S. latifolia *individual used in the initial cross was from Lyon, France, and the *S. dioica *individual from Davos, Switzerland.

### Cross-species amplification

To assess the transferability of our EST-SSR markers, we tested their amplification in four individuals each of 7 further *Silene *species. *S. dioica *and *S. diclinis *are both dioecious and close relatives of *S. latifolia*. *Silene acaulis*, *S. ciliata*, *S. nutans *and *S. vulgaris *are more distantly related, gynodioecious species. *Silene colpophylla *is a further dioecious species but is more distantly related to *S. latifolia*. Dioecy has evolved independently from *S. latifolia *in *S. colpophylla *[73]. Samples of these species were obtained from Davos in Switzerland for *S. dioica*, Valencia in Spain for *S. diclinis*, Leuk in Switzerland for *S. latifolia*, Davos in Switzerland for *S. acaulis*, Sierra de Guadarrama in Spain for *S. ciliata*, and Zurich in Switzerland for *S. vulgaris*. Samples of *S. nutans *were provided by P. Touzet and originated from different sites in Europe. *Silene colpophylla *samples were provided by B. Janousek and are derived from seed material originating from France.

### Linkage mapping

Linkage mapping was performed using JoinMap 4.0 [90] based on genotype data from the same 90 F2 individuals used in the segregation analysis (above). Markers with LOD scores of ≥ 3 were assigned to the same linkage group. Map distances in centiMorgans (cM) were calculated using Kosambi's mapping function. To identify linkage groups that correspond to the sex chromosomes, we used male sex as morphological marker for the Y chromosome and a microsatellite locus isolated from an X-derived BAC clone (unpublished results) to identify the X chromosome, here called linkage group 1.

## Authors' contributions

AW and MDM conceived the study and collected samples. MDM sequenced part of the cDNA library. CO and GM analyzed and annotated the sequences. MDM identified SSRs in the EST library, designed primers and tested them. AW and MDM wrote the manuscript with the support of CO and GM. All authors have read and approved the final manuscript.

## Supplementary Material

Additional file 1 **Supplemental Table 1**. Microsatellite markers developed for *Silene latifolia*. Click here for file

Additional file 2 **Supplemental Table 2**. Results of BLASTX searches of the 30 EST-SSRs against *Arabidopsis thaliana*. Click here for file
