# Supplementary material for: A White Campion (Silene latifolia) floral expressed sequence tag (EST) library: annotation, EST-SSR characterization, transferability, and utility for comparative mapping
Source: BMC Genomics. 2009 May 25;10:243. doi: 10.1186/1471-2164-10-243 (PMC2689282; doi:10.1186/1471-2164-10-243)
Supplement: Additional file 1 — Supplemental Table 1. Microsatellite markers developed for Silene latifolia. [file 1471-2164-10-243-S1.doc]

| **Supplemental table 1: Microsatellite markers developed for *Silene latifolia***  Only primer pairs amplifying reliably in *Silene latifolia* are listed. | | | | | | | | | | | |
| --- | --- | --- | --- | --- | --- | --- | --- | --- | --- | --- | --- |
| Locus | Repeat motif | Primer sequence (5’-3’) | Size range (bp) | Genomic position | No. of alleles | H_O_ | H_E_ | HWE | F_is_ | PIC | LG |
| SL_eSSR01 | (AACCCA)_4.7_ | (F) ATCCGTAAATTTCCGCCTTC  (R) GTCATGGCGATTGACTCAGA | 213-237 | CDS | 4 | 0.333 | 0.628 |  | 0.474 | 0.617 | unlinked |
| SL_eSSR02 | (TAATT)_5_ | (F) GCCGGAATTCCTTTTTGTTC  (R) ATGGTTCGATCAACCTCGTC | 210-225 | 5’ UTR | 3 | 0.074 | 0.458 | ***** | 0.841 | 0.449 | 4 |
| SL_eSSR03**^+^** | (GTA)_13_ | (F) AGCAGTGTCACCGGTTTAGG  (R)GGTTGTTGACAATTCCGGTACTA | 162-267 | 3’ UTR | - | - | - |  | - | - | 1 |
| SL_eSSR04 | (ATA)_6_ | (F) GAAGGAGCTGGATGAACTCG  (R) TTTACTTAATGATCCCATAATTCCA | 170-179 | CDS | 4 | 0.666 | 0.682 |  | -0.027 | 0.673 | 5 |
| SL_eSSR05**^+^** | (AG)_8_ | (F) TCCCAGTTGTCCGAAATACC  (R) AAATCCACGCTTGATTGGAC | 231-247 | 5’ UTR | - | - | - |  | - | - | 3 |
| SL_eSSR06 | (AAT)_9_ | (F) GAAACATTTCCCCACTCACAA  (R) AACCGGGTCACGTATTTCAG | 160-184 | 5’ UTR^1^ | 9 | 0.333 | 0.684 | ***** | 0.516 | 0.670 | 3 |
| SL_eSSR07 | (TCAAT)_9_ | (F) TCGATCAAAATCCCCAATTC  (R) AACGACGGAGAAAGAATGGA | 164-179 | 5’ UTR | 3 | 0.344 | 0.609 |  | 0.438 | 0.599 | unlinked |
| SL_eSSR08**^+^** | (TAA)_21_ | (F) GCATGAAATCATTTTTCAGAGG  (R) CGAAAAACACCACCAAAACC | 219-258 | 5’ UTR^2^ | - | - | - |  | - | - | unlinked |
| SL_eSSR09 | (CAT)_7_ | (F) AGATCCATTGGGCAAAAATG  (R) GGTAGCGAGAGAGACGATGG | 226-250 | CDS | 5 | 0.381 | 0.414 |  | 0.083 | 0.404 | 3 |
| SL_eSSR10 | (GT)_9_ | (F) CTAATCACCCGCGTTTCAAT  (R) GATCTTCTTCCGGCATTTGA | 342-360 | 5’ UTR | 9 | 0.703 | 0.823 |  | 0.147 | 0.807 | 6 |
| SL_eSSR11 | (TTA)_8.7_ | (F) TGTGGCTGCCTCAATGATTA  (R) GCTTCTCTGCTTTCAGGAACA | 225-285 | 3’ UTR | 12 | 0.416 | 0.906 | ***** | 0.542 | 0.881 | unlinked |
| SL_eSSR12 | (TC)_8_ | (F) CGTTCCTTCACCTCCACATT  (R) ATTCATGGCGGAGGTATGAG | 165-195 | 5’ UTR | 8 | 0.833 | 0.803 |  | -0.038 | 0.786 | unlinked |
| SL_eSSR13 | (AG)_11_ | (F) CCATCGCCTCCATCAATCT  (R) TGGTGCTCCATTGATCTTGA | 207-215 | 5’ UTR | 5 | 0.625 | 0.758 |  | 0.130 | 0.729 | 1 |
| SL_eSSR14 | (AGC)_9.7_ | (F) CACCACTCCACATCCTTCCT  (R) CTTTCAATCCTCTCCGCATC | 319-346 | CDS | 7 | 0.733 | 0.678 |  | -0.082 | 0.667 | 4 |
| SL_eSSR15 | (ATCAAG)_4.8_ | (F) AGCCGGCAATACACATTCTC  (R) GCCAACTCCGTCCTACCATA | 192-234 | CDS^3^ | 5 | 0.533 | 0.645 |  | 0.176 | 0.634 | - |
| SL_eSSR16 | (ATC)_5_ | (F) AACCAACACCAGCAACCTTC  (R) TTCTTTGCCACTTCTTCACTCA | 185-200 | 5’ UTR | 5 | 0.333 | 0.632 | ***** | 0.521 | 0.601 | 6 |

| **Supplemental table 1: Continued** | | | | | | | | | | | |
| --- | --- | --- | --- | --- | --- | --- | --- | --- | --- | --- | --- |
| SL_eSSR17 | (ATT)_13.3_ | (F) CCCCTTTTCTTCTCCTCCAA  (R) CACCAGTTCCTGCACAAAAC | 238-280 | 5’ UTR | 10 | 0.655 | 0.643 | ***** | -0.005 | 0.607 | unlinked |
| SL_eSSR18 | (AG)_12_ | (F) GCACAGCTTCTACTACCAACCA  (R) TCCAACCTTGGACAAACACA | 249-267 | 5’ UTR | - | - | - |  | - | - | - |
| SL_eSSR19 | (TCATC)_5_ | (F) TGAAGTCTTGTGTGCCTGCT  (R) CGTCGTTACGCCTTTTTAGC | 186-196 | CDS | 5 | 0.172 | 0.284 |  | 0.464 | 0.259 | - |
| SL_eSSR20 | (TGA)_5_ | (F) CAACAACTGAGGCGCTACAA  (R) AGCAGCTCTTTTGGGTTTGA | 194-206 | CDS | 4 | 0.192 | 0.275 |  | 0.305 | 0.270 | unlinked |
| SL_eSSR21 | (AAG)_7_ | (F) GGGCAGGGTGGTAGAACAT  (R) GCTTCGGATTTCTCCTCAAA | 236-239 | 5’ UTR | 2 | 0.500 | 0.382 |  | -0.315 | 0.375 | 1 |
| SL_eSSR22 | (ACA)_8.3_ | (F) CACCATTTCTTCACGGCTTC  (R) GCTGTTGTTAATGGCGGATT | 173-194 | CDS | 6 | 0.750 | 0.774 |  | 0.032 | 0.760 | 5 |
| SL_eSSR23 | (CCCAAT)_4.7_ | (F) TGGCGATCAAGCTTTTCTCT  (R) GGAAATTGGGGAGATTAGGG | 211-235 | CDS | 3 | 0.178 | 0.283 |  | 0.375 | 0.279 | - |
| SL_eSSR24 | (CT)_16.5_ | (F) TGCAATTTTTCTCCATCAACA  (R) CAATGAACCAAAATTGTCGAA | 195-217 | No hit | 7 | 0.320 | 0.741 | ***** | 0.573 | 0.726 | 1 |
| SL_eSSR25 | (TCA)_12.3_ | (F) CCGTTAAACGCCTTCTTCAA  (R) CATGACGTTTCCGAGGAGTT | 230-260 | CDS | 7 | 0.777 | 0.768 |  | -0.012 | 0.748 | 2 |
| SL_eSSR26**^+^** | (ATT)9 | (F) AACATGGGTGAAGGGATCAA  (R) AATTAGGTCCCGGAAATTGG | 208-268 | 5’ UTR^4^ | - | - | - |  | - | - | unlinked |
| SL_eSSR27 | (CATCCT)_6.2_ | (F) CTTTCAATGCCAGGCTCTTC  (R) GACGAGTGCGATCATCTTCA | 240-264 | CDS | 6 | 0.535 | 0.719 |  | 0.258 | 0.707 | 2 |
| SL_eSSR28 | (AGA)_5_ | (F) AGAAAAGTGGCGGACTCAAA  (R) CGCTGCTACCATTTCCATCT | 190-211 | CDS | 4 | 0.666 | 0.606 |  | -0.101 | 0.595 | 2 |
| SL_eSSR29 | (AAG)_8_ | (F) GAAAGATTCTCGACTAACACCTGA (R) GTCCAGCCAGTCTCATCTCC | 230-302 | CDS^5^ | 12 | 0.750 | 0.826 |  | 0.093 | 0.810 | 2 |
| SL_eSSR30 | (CTT)_5_ | (F)CATAATTTTCCTCTTAATTTGTCTACC  (R) ATGGGATCGTGTGGTTCTTC | 180-234 | 5’ UTR | 10 | 0.758 | 0.742 |  | -0.021 | 0.729 | - |

Forward primer (F), reverse primer (R); size range of alleles in base pairs (bp); observed (H_O_) and expected (H_E_) heterozygosities; fixation index (F_is_); polymorphic information content (PIC); linkage group (LG), ^+^ loci are duplicated; ^*^ indicates significant deviation from HWE after Bonferroni correction (P < 0.002).

Genomic position: ^1^ CDS (Prot4EST); ^2^ CDS (Prot4EST); ^3^ 5’ UTR (Prot4EST);^4^ CDS (Prot4EST); ^5^ 5’ UTR (Prot4EST).
