# Supplementary material for: A White Campion (Silene latifolia) floral expressed sequence tag (EST) library: annotation, EST-SSR characterization, transferability, and utility for comparative mapping
Source: BMC Genomics. 2009 May 25;10:243. doi: 10.1186/1471-2164-10-243 (PMC2689282; doi:10.1186/1471-2164-10-243)
Supplement: Additional file 2 — Supplemental Table 2. Results of BLASTX searches of the 30 EST-SSRs against Arabidopsis thaliana. [file 1471-2164-10-243-S2.doc]

| **Supplemental table 2: Results of BLASTX searches of the 30 EST-SSRs against *Arabidopsis thaliana*** | | | |
| --- | --- | --- | --- |
| Locus | Annotation | Ath Gene ID | E value / Identities |
| SL_eSSR01 | SOUL-1 binding | At2G37970 | 3e-45 / 60% |
| SL_eSSR02 | Hypothetical protein | At4G09830 | 8e-60 / 61% |
| SL_eSSR03 | EMBRYO DEFECTIVE 2001 | At2G22870 | 9e-44 / 75% |
| SL_eSSR04 | Hypothetical protein | At1G29195 | 8e-31 / 61% |
| SL_eSSR05 | RALFL22 | At3G05490 | 2e-27 / 61% |
| SL_eSSR06 | ERF domain protein | At5G44210 | 1e-19 / 79% |
| SL_eSSR07 | BASIC REGION/LEUCINE ZIPPER MOTIF 53 | At3G62420 | 3e-25 / 59% |
| SL_eSSR08 | BRANCHED-CHAIN ALPHA-KETO ACID  DECARBOXYLASE E1 BETA SUBUNIT | At1G55510 | 2e-40 / 86% |
| SL_eSSR09 | Hypothetical protein | At4G15470 | 9e-62 / 66% |
| SL_eSSR10 | GERANYLGERANYLATED PROTEIN | At4G24990 | 5e-47 / 72% |
| SL_eSSR11 | Hypothetical protein | At1G47960 | 2e-24 / 36% |
| SL_eSSR12 | ARABIDOPSIS RAS 5 | At1G02130 | 6e-93 / 90% |
| SL_eSSR13 | 40S ribosomal protein S16 | At2G09990 | 3e-68 / 88% |
| SL_eSSR14 | Hypothetical protein | At5G04440 | 3e-71 / 68% |
| SL_eSSR15 | Ribosomal protein S4 | ArthMp027 | 2e-18 / 47% |

| **Supplemental table 2: Continued** | |  |  |
| --- | --- | --- | --- |
| SL_eSSR16 | DNA-Binding Protein | At2G45820 | 1e-11 / 66% |
| SL_eSSR17 | MSP1 protein / intramitochondrial sorting protein | At4G27680 | 1e-68 / 85% |
| SL_eSSR18 | Hypothetical protein | At2G17240 | 2e-19 / 91% |
| SL_eSSR19 | Hypothetical protein | At3G49570 | 6e-07 / 51% |
| SL_eSSR20 | ARABIDOPSIS THALIANA NUDIX HYDROLASE HOMOLOG 15 | At1G28960 | 6e-52 / 58% |
| SL_eSSR21 | Hypothetical protein | At1G69980 | 4e-17 / 40% |
| SL_eSSR22 | COATOMER PROTEIN EPSILON SUBUNIT FAMILY PROTEIN | At1G30630 | 2e-99 / 78% |
| SL_eSSR23 | Hypothetical protein | At1G65720 | 1e-09 / 63% |
| SL_eSSR24 | No hit | - | - |
| SL_eSSR25 | Hypothetical protein | At1G19140 | 2e-44 / 74% |
| SL_eSSR26 | SKS11 (SKU5 Similar 11) | At3G13390 | 6e-73 / 75% |
| SL_eSSR27 | No hit | - | - |
| SL_eSSR28 | Hypothetical protein | At3G09980 | 3e-43 / 46% |
| SL_eSSR29 | ARABIDOPSIS BLUE-COPPER-BINDING PROTEIN | At5G20230 | 1e-10 / 39% |
| SL_eSSR30 | Hypothetical protein | At5G66230 | 2e-15 / 62% |
